# Evaluation of corticoresistance in patients with thyroid eye disease and use of rituximab as a second-line treatment

**DOI:** 10.1007/s12020-024-04108-4

**Published:** 2024-11-28

**Authors:** Klara Pekarova, Jan Schovanek, Roman Dohnal, Martin Radvansky, David Karasek, Marta Karhanova

**Affiliations:** 1 Department of Internal Medicine III—Nephrology, Rheumatology and Endocrinology, Faculty of Medicine and Dentistry, Palacky University Olomouc and University Hospital Olomouc, Olomouc, Czech Republic; 2 Department of Computer Science, Faculty of Electrical Engineering and Computer Science, VSB Technical University of Ostrava, Ostrava, Czech Republic; 3 Department of Ophthalmology, Faculty of Medicine and Dentistry, Palacky University Olomouc and University Hospital Olomouc, Olomouc, Czech Republic

**Keywords:** thyroid eye disease, clinical activity score, immunosuppressive treatment, corticoresistance, corticosensitivity, rituximab

## Abstract

**Purpose:**

High-dose intravenous glucocorticoids are the standard first-line treatment in active, moderate to severe and severe thyroid eye disease (TED). We evaluate the usefulness of clinical activity score (CAS) and thyroid-stimulating immunoglobulin (TSI) as predictors and/or post-treatment markers of corticoresistance in patients with TED and the effect of rituximab in second-line treatment.

**Methods:**

We enrolled 236 patients with an active TED into this retrospective single-tertiary-center cohort study. All patients were initially treated with high-dose systemic glucocorticoids. Rituximab was later administered to 29 of 42 corticoresistant patients.

**Results:**

The CAS of the corticoresistant patients was significantly higher both before (*p* = 0.0001) and after (*p* = <0.0001) first-line treatment compared to the corticosensitive group. ROC analysis established the cut-point value as CAS ≥ 2.5 with a sensitivity of 96.3%, specificity of 57.5% and area under the curve of 82.8%. In 22 patients treated with rituximab, CAS gradually decreased to zero values without reactivation during extended follow-up. There was no difference in the TSI of corticosensitive and corticoresistant patients before or after first-line therapy.

**Conclusion:**

CAS ≥ 2, after first-line treatment, could be used as a corticoresistance marker. Corticoresistant patients should be subject to long-term follow-up for early detection of reactivation to reduce the delay to second-line treatment. Rituximab is a well-tolerated choice of second-line treatment and has a long-lasting effect on disease activity. Although TSI is a valuable biomarker of Graves’ disease and TED activity, according to our results, TSI cannot be used as a marker of corticoresistance.

## Introduction

Thyroid eye disease (TED) is a progressive autoimmune inflammatory disease of soft orbital tissues closely related to thyroid autoimmunity. Orbital and periorbital changes can lead to visual impairment, limitation of eye movement, diplopia and appearance changes. These factors significantly impact the QoL (quality of life) of TED patients, affecting their visual function and appearance [1, 2]. The time of TED presentation differs among patients [3, 4]. The clinical activity score (CAS) evaluates the disease activity in everyday clinical practice. Its characteristics make CAS easily assessed by clinical examination without requiring laboratory or imaging methods or special medical equipment [5]. EUGOGO classifies the severity of the disease into three degrees: mild, moderate to severe, and sight-threatening (including DON and corneal breakdown) [6, 7]. One of the TED activity biomarkers is the level of TSH receptor antibodies (TRAb). Its stimulating fraction, thyroid-stimulating immunoglobulin (TSI, or stimulatory TSH-R antibodies, TSAb), is ideally detected by cell-based bioassays, which can differentiate the functionality type of the antibodies (stimulating, neutral or blocking). This method is not routinely available, and so-called “bridging” immunoassays are commonly used [8, 9]. The standard first-line treatment for active, moderate to severe and severe TED are high-dose intravenous glucocorticoids (ivGC), sometimes also combined with mycophenolate-mofetil [10]. Some studies also effectively used orally administered glucocorticoids combined with mycophenolate mofetil [11]. Unfortunately, up to 20–25% [12] of clinically active, moderate-to-severe TED patients either do not respond to standard first-line treatment or their TED reactivates sometime after treatment termination. In these corticoresistant (CR) patients, a chimeric mouse/human antibody, rituximab (RTX), is listed in the EUGOGO guidelines as a valid second-line treatment [6]. However, dosing schemes vary among authors [6, 13–19]. Monoclonal antibodies directed against IGF-1R Teprotumumab, the first-line and only FDA-approved treatment for TED, are currently unavailable in Europe [20]. Another option with a documented effect on eye muscle motility and/or diplopia is orbital radiotherapy combined with glucocorticoids [6].

In urgent states, like DON or severe corneal exposure, not responding to ivGC and any eyeball subluxation is the surgery (orbital decompression) inevitable choice. Otherwise, to correct strabismus, proptosis, lid malposition or chronic orbital congestion, surgery should be postponed to disease activity disappearance [20].

Early recognition of CR patients could minimize the time gap between the first- and second-line treatment, enabling them to achieve the highest possible quality of life. In this study, we evaluate the usefulness of CAS and TSI as predictors and/or post-treatment markers of corticoresistance in patients with TED.

## Materials and methods

In this retrospective single-tertiary-center cohort study, we included 236 patients (159 women and 77 men, aged 18–85 years) with active moderate-to-severe TED from our register, all of whom were initially treated with high-dose systemic corticosteroids (methylprednisolone) between 2007 and 2022.

Patients with an initially sight-threatening TED requiring acute orbital decompression were excluded from the study. The demographic and clinical data are presented in Table 1. ivGC administration was performed during three short hospitalizations over three months. Patients obtained different cumulative doses of 7.5 g, 6 g and 4.5 g, and singularly different doses of ivGC as the EUGOGO recommendations changed over the time period of the study. In between the hospitalizations, patients received decreasing doses of oral prednisone (tapering from 20 mg daily).

**Table 1:** Patient information

Variables	Corticosensitive patients	Corticoresistant patients	Difference between CS and CR variables (*p* value)
**Number of patients**	194	42	–
**Gender (female)**	134 (69%)	25 (59%)	0.2763
**Age at first-line treatment**	53 years	54.5 years	0.9493
**Cumulative dose of ivGC**	7.5 g: 152, 6 g: 33, 4.5 g: 9	7.5 g: 22, 6 g: 10, 4.5 g: 3	0.4122
**Graves‘ disease**	131 (68%)	27 (64%)	0.3700
**Smoker before treatment (active or passive)**	107 (55%)	29 (69%)	0.1214
**Smoker during treatment (active or passive)**	30 (16%)	4 (10%)	0.3513
**Lateralization (bilateral/right/left)**	bilateral: 151, right: 21, left: 22	bilateral: 35, right: 3, left: 4	0.4825
**Duration of Thyroid disease before TED (days)**	median: 153, average: 1404	median: 122.5, average: 1038	0.6269
**Duration of TED prior treatment (ivGC) (days)**	median: 163, average: 453	median: 124 average: 507	0.1959
**Total Thyroidectomy (TTx)**	171 (88%)	33 (79%)	0.1270
**Radioiodine post TTx (TTx contraindicated/Radioiodine ablation of Thyroid Remnants)**	91 (47%)	7 (17%)	**0.0031**
**Decompression**	5 (3%) (as a form of corrective surgery)	in 2 (5%) as a form of corrective surgery, in 22 (52%) as a treatment option	**<0.0001**

Bold values are the values that are statistically significant according to the significance level 0.05 (that is, <0.05).

In our study, we use the term “corticosensitive” for patients whose response to first-line treatment was complete (objective/subjective significant change in activity) – no signs of reactivation were observed, and no other medical intervention due to active disease was needed. The term “corticoresistant” is used when the response to first-line treatment was incomplete, or TED reactivated and medical intervention was needed. RTX was administered intravenously to 29 CR patients during short hospitalization in a single dose of 100 mg, which was repeated if clinically needed. Twenty-five patients received a cumulative dose of 100 mg, two had a cumulative dose of 200 mg, and two had a cumulative dose of 400 mg of RTX. Off-label use of rituximab was reported to the State Institute for Drug Control. Alternatively, to report the treatment outcomes, we applied the EUGOGO composite index on our CAS and diplopia (muscle duction) data [6].

**Fig. 1:**
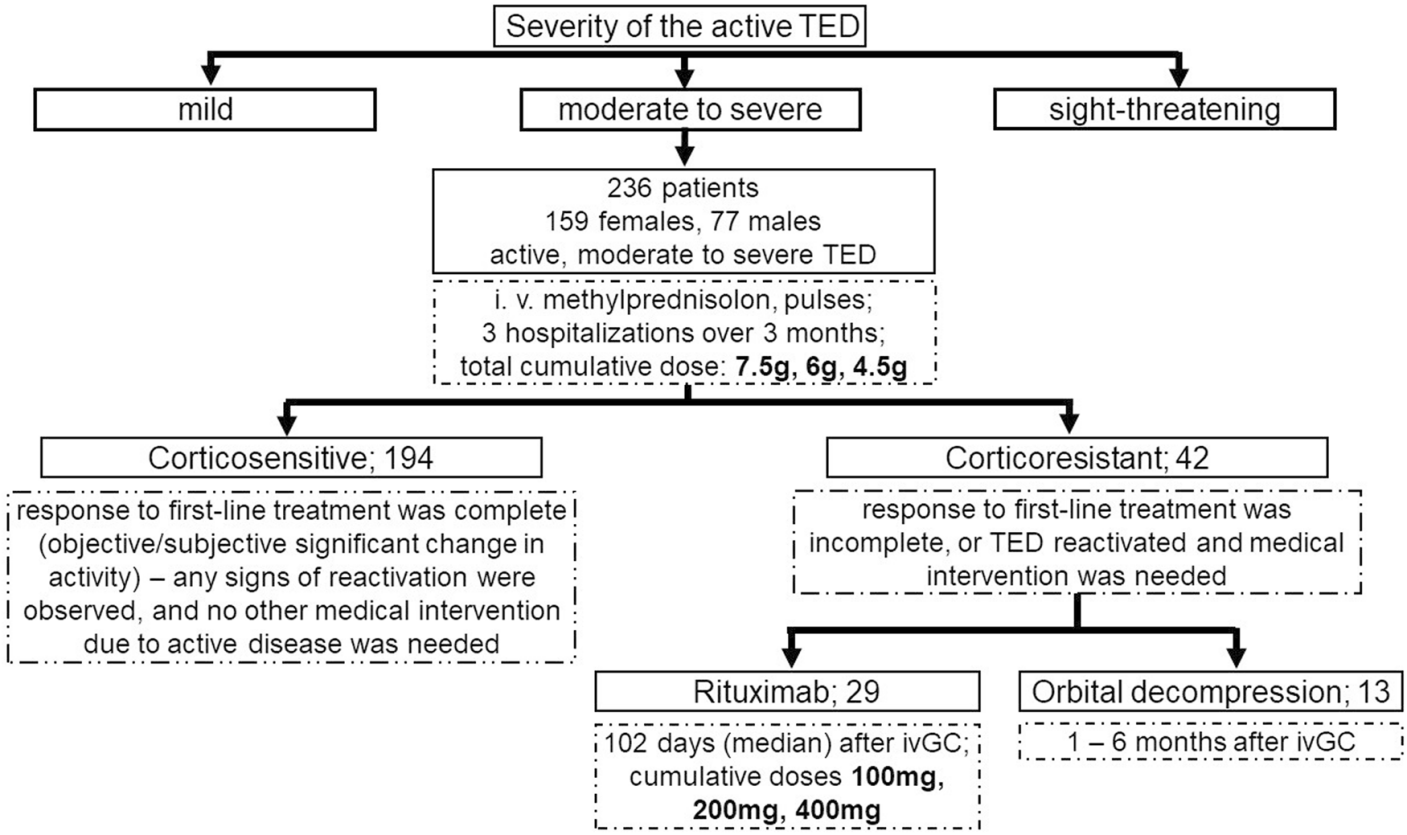
Scheme of patient subgroup distribution and treatment sequence

After both first- and second-line treatments, all patients were educated about the signs and symptoms of TED reactivation. The schematization of retrograde patient recruitment and their distribution into subgroups is summarized in Fig. 1. Biochemical analyses were performed from peripheral venous blood samples obtained at admission, before hospital discharge and at scheduled checkups. Thyroid-stimulating immunoglobulin (TSI, IU/L) levels were determined immunologically (LEIA) using the Immulite 2000 analyzer (Siemens Healthineers, Germany). Exophthalmos was measured using a Hertel Exophthalmometer, while the same clinical ophthalmologist evaluated diplopia and disease activity by the standardized 7-point Clinical Activity Score (CAS) scale [6]. All data was expressed as mean ± standard deviation (SD) or median and interquartile range (IQR), as specified. For data analyses, both parametric and non-parametric methods were used. The Shapiro-Wilk test was utilized to test the data distribution. Paired T-tests, Mann-Whitney paired tests and receiver operating characteristics (ROC) analyses were performed as appropriate. Statistical significance was set as *p* < 0.05. All tests were performed, and figures were created using GraphPad Prism 8.4.3.686 for Windows (San Diego, California, USA).

## Results

### Demographic

The study population includes all consecutive patients treated at our center who met the inclusion criteria. All patients received ivGC as their first treatment for TED. Some had previously received minor doses of oral methylprednisolone before being referred to our center; however, these data were not systematically recorded in our system. Of 194 CS patients, 69% were women aged 51.5 ± 13.1 years; the CR group comprised 42 patients, of which 60% were women aged 51.7 ± 14.1 years; the groups did not differ in gender and age at first-line treatment, lateralization of TED, cumulative dose of ivGC administered and smoking status. 55% of CS patients were smokers before the start of the treatment, and 15% were smoking at the end; in the CR group there were 72.5% smokers before, and 9.5% during the treatment. Graves’ disease had 131 CS patients (68%) and 27 (64%) CR patients. The groups neither differed in the duration of TED before first-line treatment nor the time from diagnosis of thyroid disease and TED development. As a euthyroidism restoring therapy, most of the patients were referred to total thyroidectomy - TTx underwent 171 (88%) CS patients and 33 (79%) CR patients. Thyroid gland radioiodine ablation was used as a part of euthyroidism restoring therapy in patients where TTx was contraindicated (in 6 patients from the CS group) or was used as an additional treatment after TTx (in 85 from CS and 7 from CR group) - as a Radioiodine ablation of Thyroid Remnants [21], significantly more patients underwent thyroid gland radioiodine ablation from the CS group (*p* = 0.0031). In the rest of the patients, euthyroidism was restored by peroral treatment (thiamazole). Euthyroidism was restored in all of our patients.

Decompressive surgery was performed in 5 CS (due to chronic non-active exophthalmos more than six months after ivGC) and 24 CR patients; of these, 13 patients were indicated to urgent decompression (due to severe TED, and those did not receive RTX); 11 CR patients were in second-line treated with RTX, whereby decompression was performed in two of them as a form of corrective surgery due to chronic non-active exophthalmos, four of them in between ivGC and RTX, and five of them within six months from the last RTX dose due to absence of clinical response or reactivation (RTX resistant/nonsensitive). The group characteristics are summarized in Table 1.

### Effect of first-line treatment

The median CAS of the CS patients dropped after first-line treatment from 3 (IQR: 2–4) to 1 (IQR: 0–1); in CR patients it dropped from 4 (IQR: 3–5) to 3 (IQR: 1–4). We found that the CR patient CAS was significantly higher both before (*p* = 0.0001) and after (*p* = < 0.0001) first-line treatment when compared to the CS group.

**Fig. 2:**
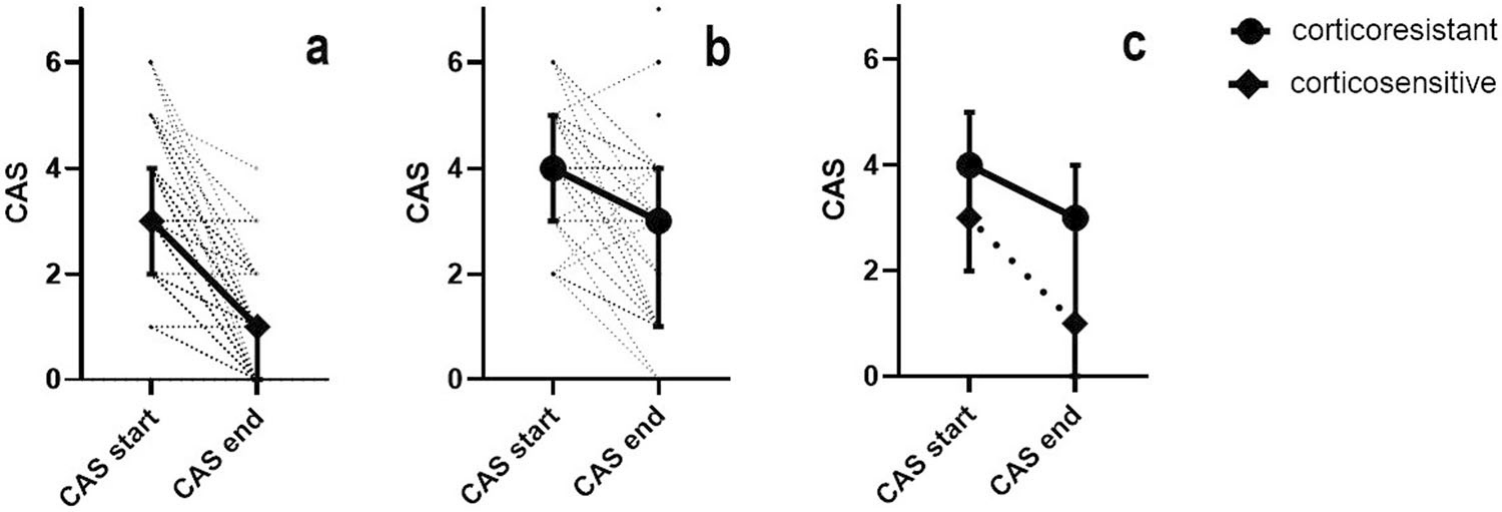
Graphs of CAS evolution after first-line treatment, start = before first-line treatment, end = after first-line treatment, (**a**) – CS patients, (**b**) – CR patients, (**c**) – CS and CR patients (median and interquartile range)

ROC analysis of the CAS at the end of first-line treatment in CR and CS patients computed a CAS cut point value of 2.5 with a sensitivity of 96.3%, specificity of 57.5% and area under the curve 82.8% with a positive predictive value of 0.915. For detailed CAS values see Fig. 2.

In CS patients, CAS did not correlate with the TED duration prior to the treatment, r = −0.07 (*p* = 0.346), nor after the end of first-line treatment, r = −0.111 (*p* = 0.129). Similarly, in CR patients, CAS did not correlate with the duration of TED prior to the treatment, r = −0.196 (*p* = 0.252), nor after the end of first-line treatment, r = −0.232 (*p* = 0.15).

In the group of CS patients, there was a significant decrease in diplopia, from 58 to 23 patients out of 100 (*p* < 0,0001); on the other hand, in the group of CR patients, diplopia improved only in 2 patients after the first line treatment, while only in 2 was not present at all (*p* = 0,6671). Although Hertel of the CS patients reduced after first-line treatment and Hertel of the CR patients increased after the first-line treatment, these results are unfortunately not statistically significant; the change was within 1 mm in both groups. Data are displayed in Table 2.

**Table 2:** Diplopia and proptosis—descriptive statistics (OD = right eye, OS = left eye)

	Corticosensitive patients		Corticoresistant patients			Corticosensitive patients				Corticoresistant patients			
	Diplopia start ivGC	Diplopia end ivGC	Diplopia start ivGC	Diplopia end ivGC		Hertel OD start ivGC (mm)	Hertel OD end ivGC (mm)	Hertel OS start ivGC (mm)	Hertel OS end ivGC (mm)	Hertel OD start ivGC (mm)	Hertel OD end ivGC (mm)	Hertel OS start ivGC (mm)	Hertel OS end ivGC (mm)
**Number of patients**	58 out of 100	23 out of 100	23 out of 25	21 out of 25	**Median**	20	20	20	19.5	21.75	22	21	21.25
**Mean**	0.58	0.23	0.92	0.84	**Mean**	20.62	20.23	19.88	19.68	21.61	21.94	21.83	21.9
**Std. Deviation**	0.496	0.423	0.2769	0.3742	**Std. Deviation**	3.511	3.48	3.583	3.535	3.622	3.35	3.406	3.519
**Std. Error of Mean**	0.0496	0.0423	0.05538	0.07483	**Std. Error of Mean**	0.2588	0.2565	0.2641	0.2606	0.6036	0.5584	0.5676	0.5865

### Effect of second-line treatment

In the 29 patients who, as a second-line treatment, obtained low dose RTX (RTX subgroup), the median time between the end of first-line treatment (ivGC) and reactivation (RTX dose) was 96 days (IQR: 52.5–261.5). 69% were women, 51.61 ± 14.6 years of age, 65.5% of which smoked before and 7% of which during the treatment, which is not different from the CS group. Eleven of them underwent decompression, as explained above.

**Fig. 3:**
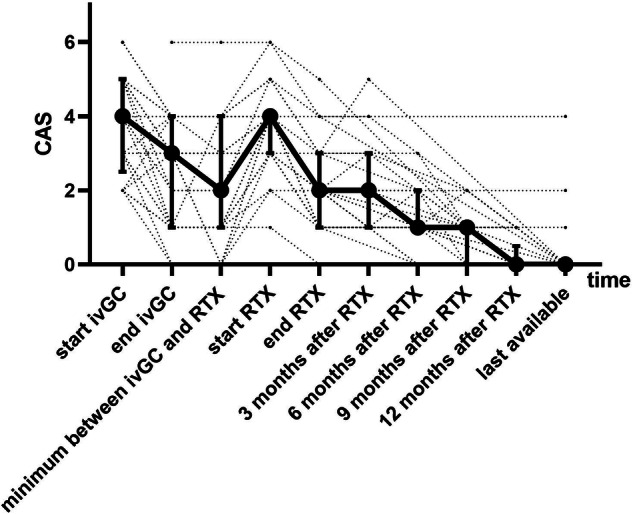
CAS evolution over time in second-line Rituximab-treated patients

In 22 patients treated by RTX, CAS gradually decreased to zero values without any signs of reactivation during extended follow-up (median 832 days, IQR: 420.5–1455) (Fig. 3). TED reactivated in seven patients after RTX in a median of 114 days (IQR: 45–1014). There was no difference in the rate of reactivations after RTX from the rate after ivGC (*p* = 0.81).

TSI of CR and RTX-treated patients dropped from a median of 4.89 (IQR: 0.66–22.3) to 2.31 (IQR: 0.36–8.44) after first-line treatment (*p* = 0.22) and later to 1.86 (IQR: 0.1–7.41) after RTX (*p* = 0.12). TSI of the CS subgroup dropped from a median of 3.64 (IQR: 0.95–8.13) to 1.29 (IQR: 0.38–3.37) (*p* = 0.0034). There was no significant difference between the TSI of the CS and CR patients before (*p* = 0.46) or after first-line therapy (*p* = 0.56).

## Discussion

Evaluating corticoresistance is critical both before and after first-line treatment for patients predicted to be CR to avoid the potential side effects of first-line therapy and delays in providing effective therapy. Furthermore, CR patients should be offered second-line treatment as early as possible. We wish to offer our patients the chance to maintain as high a quality of life as possible by minimizing the time between TED development and effective treatment. Moreover, possible adverse events of each treatment type could be avoided.

The current EUGOGO guidelines recommend assessing treatment efficacy through both subjective (patient-reported outcomes) and objective (clinician-reported outcomes) primary measures. A recently revised composite index has also been proposed [6]. When we applied this index to our data, patients deemed corticoresistant would be classified as “non-responders” based on EUGOGO parameters, which consider a combination of CAS and diplopia data from our dataset (data not shown). Perez-Moreiras et al. use the term “corticosteroid resistant” for patients with incomplete response (defined as a CAS improvement < 2) to at least three doses of 500 mg of ivGC or recurrence of TED, defined as an increase in CAS ≥ 1 after treatment with ivGC [22]. We found that the CR patients’ CAS was significantly higher before and after the first-line treatment than the CS group. Performed ROC established the cut-point value as CAS was ≥ 2.5 with a sensitivity of 96.3%, specificity of 57.5% and area under the curve of 82.8%. Therefore, we suggest CAS ≥ 2 after first-line treatment as a possible marker of corticoresistance and that patients with higher CAS should be subject to regular long-term follow-up. Early recognition of the ineffectiveness of the first-line treatment or reactivation of the disease could enable early access to the second-line treatment. It is essential to follow up high-risk patients after successful therapy and, in case of reactivation, offer them personalized additional treatment based on their medical and personal history and preferences to maintain as high quality of life as possible with the best clinical outcome. When evaluating TSI, another possible marker of corticoresistancy in our cohort, we did not find a significant difference between CS and CR patients.

Mourits, Prummel et al. showed that the outcome of glucocorticoid treatment was unrelated to the total duration of TED [5, 23, 24], which is also true for rituximab treatment [17]. Similarly, we did not find any correlation between the length of TED prior to treatment and CAS at the beginning or end of first-line treatment. Moreover, CS and CR patients did not differ in the length of TED before first-line treatment.

TSI of the CR patients treated with RTX in the second-line did not change significantly, although it dropped after first-line treatment and after RTX; none of these decreases were significant from the pre-treatment value. The TSI of the CS subgroup dropped significantly after first-line therapy. CR and CS groups did not differ in TSI values. Although TSI is a valuable, highly sensitive biomarker of Graves’ disease and TED activity, according to our results, TSI cannot be used as a marker of corticoresistance. However, given that TSI values were not available for all time points in our cohort, we can assume that if data from larger samples was analyzed, the statistical significance and strength of evidence could differ [25–27].

We found a higher distribution of patients treated by radioiodine in the CS than in the CR. However, radioactive iodine usage and oxidative stress are relevant risk factors for TED development and progression. We supposed this difference was mainly caused by the recruitment of patients over a long time while the guidelines on TED treatment and thyroid gland dysfunction were changing. Several studies reported the long-term positive effect of RTX on TED [28]. In the second part of our study, we followed 29 corticoresistant patients who reactivated after first-line treatment in the median of 96 days. Low-dose RTX (100 mg) was reported to be effective as TED first-line treatment [15], but also as a second-line treatment in previously corticoresistant TED patients [29]. Lower doses lead to a reduction in the risk of potential side effects. One of the reported potential adverse severe events is DON, which must be monitored and treated appropriately. While treating our CR patients with low-dosed RTX as second-line treatment (a single dose was 100 mg per cycle), we did not observe any significant side effects, and RTX was well tolerated in all of them.

It should be noted that four of them were after orbital decompression before RTX. As one of the main goals of this study, we focused on CAS evolution over time in TED patients. As mentioned, CAS was significantly higher in RTX-treated patients before and after first-line treatment. We could see the long-term positive effect of RTX documented by the gradual, significant and long-term decrease of CAS to zero values.

In patients who do not sufficiently respond to RTX as a second-line treatment and show high activity of the disease and/or several eye and sight impairments, urgent orbital decompression should be considered [6, 30]. From our RTX subgroup, two patients underwent decompression as a form of corrective surgery due to chronic non-active exophthalmos, five due to the absence of clinical response or reactivation, and four in between the treatment cycles. Decompression as a corrective surgery due to chronic non-active exophthalmos was performed in five CS patients. Urgent orbital decompression must be performed in the event of DON cases unresponsive to ivGC [2, 6]. Thirteen CR patients were indicated for decompression (less than six months from the end of treatment) due to the persisting activity and severity of the disease after first-line treatment.

This study has several limitations. As we serve as a referral center, we included TED patients treated with ivGC in other hospitals, some of whom were administered “unusual” cumulative doses of ivGC. Due to our study’s retrospective design, the observed effect of rituximab could be affected by the delayed effect of previous treatment or by the natural course of the disease described by Rundle [31]. Also, we could not provide a predictive solution for corticoresistency before initiating ivGC. On the contrary, this sample represents the largest cohort of patients describing sequentially the effect of first- and second-line treatment in the same patients and, therefore, describing TED development over time as we see in our real-life patients.

Based on our data, CR patients could be detected by CAS score after first-line treatment ≥ 2. These patients should be subject to long-term follow-up for early detection of reactivation. Timely diagnosis would allow them early access to second-line treatment and enhance their chances of reaching the best treatment effect and final quality of life. RTX is a well-tolerated choice of second-line treatment and, even in low doses, has a long-lasting effect on disease activity, as documented by the progressive and long-term decrease of CAS. Nevertheless, further research is still needed to find markers of corticoresistance prior to ivGC.

## Supplementary information

Supplementary figure legend

Supplementary figure

## Data Availability

No datasets were generated or analysed during the current study.
