# Supplementary material for: Evaluation of corticoresistance in patients with thyroid eye disease and use of rituximab as a second-line treatment
Source: Endocrine. 2024 Nov 28;87(3):1112–9. doi: 10.1007/s12020-024-04108-4 (PMC11845400; doi:10.1007/s12020-024-04108-4)
Supplement: Supplementary file 1 — Supplementary figure legend [file 12020_2024_4108_MOESM1_ESM.docx]

Legend to the supplementary file

Supplementary file 1: Figures of corticosensitive and corticoresistant patients before and after first line treatment (ivGC), **(a)** – corticosensitive patient before ivGC, **(b)** – corticosensitive patient after ivGC, **(c)** – corticoresistant patient before ivGC, **(d)** – corticoresistant patient after ivGC
