# Supplementary figures and images for: Evaluation of corticoresistance in patients with thyroid eye disease and use of rituximab as a second-line treatment
Source: Endocrine. 2024 Nov 28;87(3):1112–9. doi: 10.1007/s12020-024-04108-4 (PMC11845400; doi:10.1007/s12020-024-04108-4)

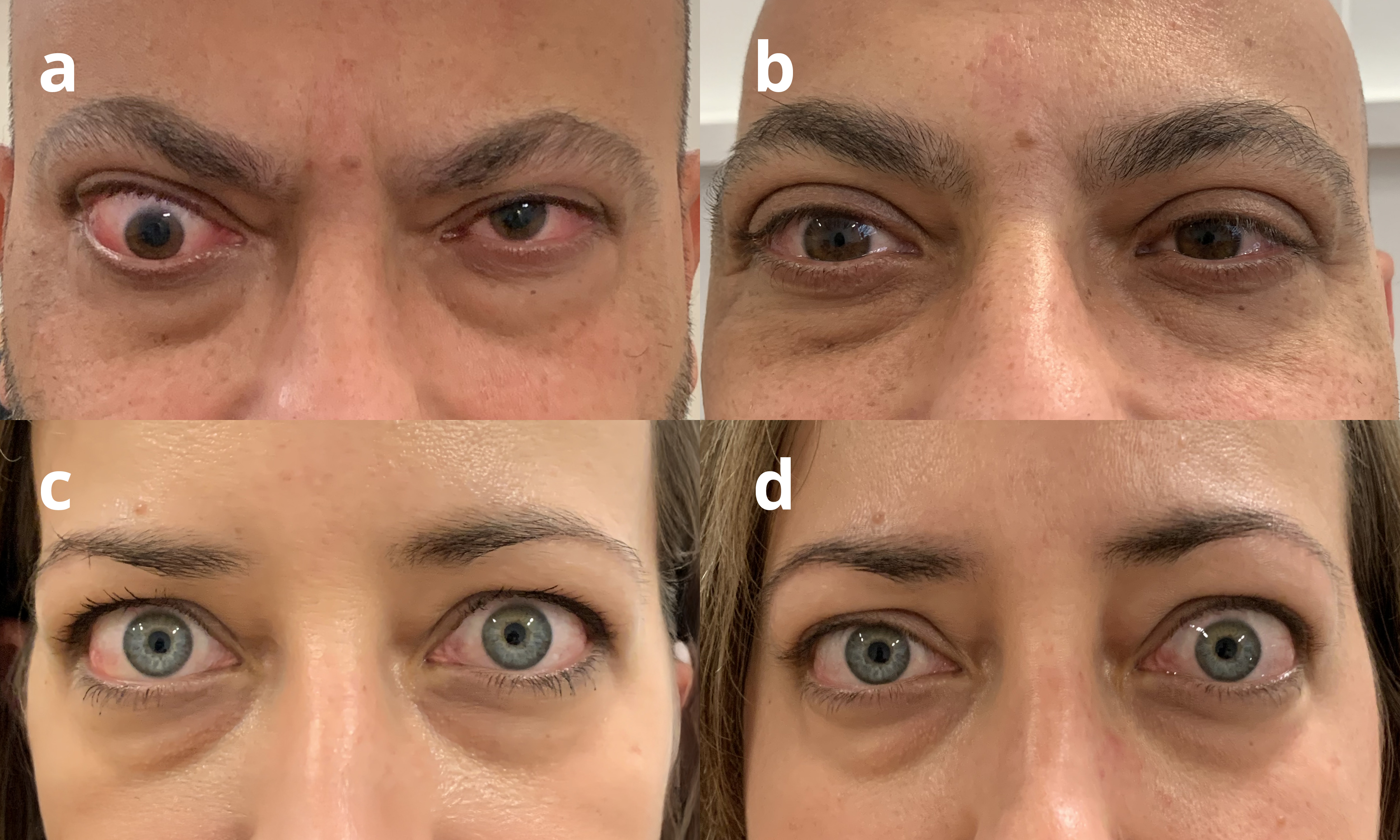

Supplement: Supplementary file 2 — Supplementary figure [file 12020_2024_4108_MOESM2_ESM.jpg]
